# Dietary fatty acids and endometrial cancer risk within the European Prospective Investigation into Cancer and Nutrition

**DOI:** 10.1186/s12885-023-10611-0

**Published:** 2023-02-16

**Authors:** S. G. Yammine, I. Huybrechts, C. Biessy, L. Dossus, S. Panico, M. J. Sánchez, V. Benetou, R. Turzanski-Fortner, V. Katzke, A. Idahl, G. Skeie, K. Standahl Olsen, A. Tjønneland, J. Halkjaer, S. Colorado-Yohar, A. K. Heath, E. Sonestedt, H. Sartor, M. B. Schulze, D. Palli, M. Crous-Bou, A. Dorronsoro, K. Overvad, A. Barricarte Gurrea, G. Severi, R. C.H. Vermeulen, T. M. Sandanger, R. C. Travis, T. Key, P. Amiano, B. Van Guelpen, M. Johansson, M. Sund, R. Tumino, N. Wareham, C. Sacerdote, V. Krogh, P. Brennan, E. Riboli, E. Weiderpass, M. J. Gunter, V. Chajès

**Affiliations:** 1 Université Sorbonne Paris Nord and Université Paris Cité, INSERM, INRAE, CNAM, Center of Research in Epidemiology and StatisticS (CRESS) , Nutritional Epidemiology Research Team (EREN), F-93017, Bobigny, France; 2 International Agency for Research on Cancer, World Health Organization, Lyon, France; 3 Dipartimento di medicina clinica e chirurgia, Federico II University, Naples, Italy; 4 Escuela Andaluza de Salud Pública (EASP), Granada, Spain; 5 Instituto de Investigación Biosanitaria ibs. GRANADA, Granada, Spain; 6 Centro de Investigación Biomédica en Red de Epidemiología y Salud Pública (CIBERESP), Madrid, Spain; 7 Department of Preventive Medicine and Public Health, University of Granada, Granada, Spain; 8 Department of Hygiene, Epidemiology and Medical Statistics, School of Medicine, National and Kapodistrian University of Athens, Athens, Grèce; 9 The German Cancer Research Center (DKFZ), Heidelberg, Germany; 10 Department of Clinical Sciences, Obstetrics and Gynecology, Umeå University, Umeå, Sweden; 11 Danish Cancer Society Research Center, Copenhagen, Denmark; 12 Department of Public Health, Faculty of Health and Medical Sciences, University of Copenhagen, Copenhagen, Denmark; 13 Department of Epidemiology, Murcia Regional Health Council, IMIB-Arrixaca, Murcia, Spain; 14 Research Group on Demography and Health, National Faculty of Public Health, University of Antioquia, Medellín, Colombia; 15 Department of Epidemiology and Biostatistics, School of Public Health, Imperial College, London, United Kingdom; 16 Department of Clinical Sciences Malmö, Lund University, Sweden; 17 Department of Molecular Epidemiology, German Institute of Human Nutrition Potsdam- Rehbruecke, Nuthetal, Germany; 18 Institute of Nutritional Science, University of Potsdam, Potsdam, Germany; 19 Cancer Risk Factors and Life-Style Epidemiology Unit, Institute for Cancer Research, Prevention and Clinical Network (ISPRO), Florence, Italy; 20 Unit of Nutrition and Cancer, Cancer Epidemiology Research Program, Catalan Institute of Oncology (ICO) - Bellvitge Biomedical Research Institute (IDIBELL). L’Hospitalet de Llobregat, 08908 Barcelona, Spain; 21 Department of Epidemiology, Harvard T.H. Chan School of Public Health, 02115 Boston, MA USA; 22 Ministry of Health of the Basque Government, Sub-Directorate for Public Health and Addictions of Gipuzkoa, San Sebastian, Spain; 23 Department of Public Health, Aarhus University, Aarhus, Denmark; 24 Navarra Public Health Institute, Pamplona, Spain; 25 Navarra Institute for Health Research (IdiSNA), Pamplona, Spain; 26 Cancer Epidemiology Centre, Cancer Council Victoria, Melbourne, VIC Australia; 27 Centre for Research in Epidemiology and Population Health, INSERM U1018, Université Paris-Saclay, Villejuif, France; 28 Human Genetics Foundation, Turin, Italy; 29 Julius Center for Health Sciences and Primary Care, University Medical Centre Utrecht, Utrecht, The Netherlands; 30 Institute for Risk Assessment Sciences (IRAS), Department of Population Health Sciences, Utrecht University, Utrecht, The Netherlands; 31 Faculty of Health Sciences, Department of Community Medicine, UiT The Arctic University of Norway, N - 9037 Tromsø, Norway; 32 Cancer Epidemiology Unit, Nuffield Department of Population Health, University of Oxford, Oxford, United Kingdom; 33 Centre for Epidemiology and Biostatistics, Melbourne School of Population and Global Health, The University of Melbourne, Melbourne, Victoria Australia; 34 Wallenberg Centre for Molecular Medicine, Umeå University, Umeå, Sweden; 35 Department of Surgical and Perioperative Sciences, Umeå University, Umeå, Sweden; 36 Cancer Registry and Histopathology Department, Provincial Health Authority (ASP 7), Ragusa, Italy; 37 MRC Epidemiology Unit, University of Cambridge, Cambridge, England, U.K.; 38 Unit of Cancer Epidemiology, Città della Salute e della Scienza University-Hospital, Via Santena 7, 10126 Turin, Italy; 39 Epidemiology and Prevention Unit, Fondazione IRCCS Istituto Nazionale Dei Tumori Di, Milano, Italy

**Keywords:** Fatty acids, Endometrial cancer, Epidemiology, Diet

## Abstract

**Background:**

Diet may impact important risk factors for endometrial cancer such as obesity and inflammation. However, evidence on the role of specific dietary factors is limited. We investigated associations between dietary fatty acids and endometrial cancer risk in the European Prospective Investigation into Cancer and Nutrition (EPIC).

**Methods:**

This analysis includes 1,886 incident endometrial cancer cases and 297,432 non-cases. All participants were followed up for a mean of 8.8 years. Multivariable Cox proportional hazard models were used to estimate hazard ratios (HR) and 95% confidence intervals (CI) of endometrial cancer across quintiles of individual fatty acids estimated from various food sources quantified through food frequency questionnaires in the entire EPIC cohort. The false discovery rate (q-values) was computed to control for multiple comparisons.

**Results:**

Consumption of n-6 γ-linolenic acid was inversely associated with endometrial cancer risk (HR comparing 5th with 1st quintile_Q5−Q1_=0.77, 95% CI = 0.64; 0.92, p_trend_=0.01, q-value = 0.15). This association was mainly driven by γ-linolenic acid derived from plant sources (HR_per unit increment_=0.94, 95%CI= (0.90;0.98), p = 0.01) but not from animal sources (HR_per unit increment_= 1.00, 95%CI = (0.92; 1.07), p = 0.92). In addition, an inverse association was found between consumption of n-3 α-linolenic acid from vegetable sources and endometrial cancer risk (HR_per unit increment_= 0.93, 95%CI = (0.87; 0.99), p = 0.04). No significant association was found between any other fatty acids (individual or grouped) and endometrial cancer risk.

**Conclusion:**

Our results suggest that higher consumption of γ-linolenic acid and α-linoleic acid from plant sources may be associated with lower risk of endometrial cancer.

## Introduction

In 2020, 417,367 new endometrial cancer cases were diagnosed and 97,370 deaths were recorded from endometrial cancer worldwide [1]. In Europe, endometrial cancer is the fourth most common cancer and the sixth most common cause of cancer death in women [1]. Overweight and obesity, poor diet and physical inactivity have been reported to increase the risk of developing endometrial cancer [2, 3]. However, evidence on the role of specific dietary factors in endometrial cancer risk is still limited [4] and prevention strategies are needed.

Experimental studies suggest two major biologically plausible mechanisms that underlie the association between endometrial cancer risk and dietary exposure particularly with regard to saturated fatty acids (SFA), unsaturated fatty acids and cholesterol. Firstly, these dietary components can modulate the production, metabolism, and excretion of endogenous hormones, which influence the proliferation of endometrial cancer cells [5–8]. Secondly, they can influence inflammatory processes, which are important in the development of many cancer types [9] including endometrial cancer where they play a central role in the regulation of endometrial mucosa growth and shedding during the menstrual cycle [10] and endometrial repair following menstruation [11].

A nutrient-wide association study from the EPIC, the Nurses’ Health Study (NHS) and the NHSII reported a higher risk of endometrial cancer in relation to a higher intake of total fat and monounsaturated fat (MUFA), but the association was primarily driven by findings from EPIC [12]. A meta-analysis by the World Cancer Research Fund (WCRF) Continuous Update Project concluded that there was “limited” evidence for a link between endometrial cancer risk and each for intake of total fat and of saturated/animal fat [4].

Data from a dose-response meta-analysis based on epidemiological studies published up to June 2015 suggested a lack of association between total dietary fat intake and endometrial cancer risk [13]. Conversely, results from another dose-response meta-analysis, including seven cohorts and fourteen case-control studies [14], suggested that higher MUFA intake was associated with lower endometrial cancer risk, while total fat and SFA intake were associated with a higher risk of endometrial cancer in the case–control studies only. The same meta-analysis [14] found no significant association between polyunsaturated fatty acids (PUFA) or linoleic acid and endometrial cancer risk. Another meta-analysis focusing on fish intake and n-3 PUFA suggested that intake of n-3 PUFA may be inversely associated with endometrial cancer risk [15].

The heterogeneity of the results from epidemiological studies in this field and the lack of information on endometrial cancer subtypes and on the types of foods (from animal or plant sources) in these studies call for larger and more in-depth investigations in the field. We therefore analyzed the association between fatty acid intake and endometrial cancer risk, overall and by different of cancer subtype stratifications, and investigated the association of dietary sources of fatty acids (animal or plant)- on endometrial cancer risk.

## Materials & methods

### Study design

The EPIC study includes 521,330 participants recruited between 1992 and 2000 from 23 centers across 10 European countries [16]. The study design, recruitment procedures and data collection have been described previously [17]. Written informed consent was provided by all study participants. Ethical approval for this study was provided by the International Agency for Research on Cancer and the institutional review boards of the local participating EPIC centers. Briefly, dietary information, as well as socio-demographic, and lifestyle data were collected at enrolment from all participants by administration of country-specific questionnaires. Self-administered questionnaires were used in all centers, except in Spain and Ragusa (Italy), where data were collected during personal interviews. In Malmo (Sweden), a combined semi-quantitative food frequency questionnaire and 7-day dietary diary and diet interview was used.

Baseline anthropometric measurements and peripheral blood samples were also collected. Procedures for sample collection, processing and storage are described in detail elsewhere [18].

A total of 308,285 women remained in the study population after exclusion of 35,700 women who had undergone hysterectomy, 25,184 prevalent cancer cases and 4,148 subjects with incomplete follow-up data. Among the included women, 2,023 cases of endometrial cancer were identified by the end of each center’s follow-up period. Further exclusions of cases were based on their tumor morphology (n = 73), lack of completion of lifestyle or dietary questionnaire (26 cases and 2,854 non cases) or classification of the women in the top or bottom 1% of energy intake to energy requirement (38 cases and 5,968 non cases). This left a total of 1,886 cases included in the analysis. Cases were morphologically classified as type I (including adenocarcinoma (NOS), adenocarcinoma in adenomatous polyp, endometrioid adenocarcinoma (NOS), mucinous adenocarcinoma, mucin-producing adenocarcinoma, adenosquamous carcinoma or adenocarcinoma with squamous metaplasia) or Type II (squamous cell carcinoma (NOS), clear cell adenocarcinoma (NOS), mixed cell adenocarcinoma, serous cystadenocarcinoma (NOS) or papillary serous cystadenocarcinoma).

Cancer end point data was based on the latest round of follow-up received from the EPIC centers and centralized at IARC between 2014 and 2016. For each EPIC study center, closure dates of the study period were defined as the latest dates of complete and verified follow-up for both cancer incidence and vital status (dates varied between centers, between June 2008 and December 2013).

### Assessment of dietary fatty acids intake

To compile the EPIC Nutrient Database (ENDB) for the EPIC study, a highly standardized procedure was used, adopting nutrient values from ten national food composition databases of the respective EPIC countries. The in-depth process for compiling this ENDB database was described in detail elsewhere [19, 20]. To date, most of the national food composition databases from the ten respective EPIC countries do not contain nutritional values for specific fatty acids isomers. Therefore, the EPIC data was matched with fatty acids isomers using the National Nutrient Database for Standard Reference of the United States (NNDSR; further referred to as USDA table) [21]. Specific foods and recipes that were not included in the USDA were decomposed in ingredients which were available in the USDA table and amounts of fatty acids were obtained through this extra USDA matching. Groupings of FA were defined as: saturated fatty acids (SFA) (4:0, 6:0, 8:0, 10:0, 12:0, 14:0, 15:0, 16:0, 17:0, 18:0, 20:0, 22:0, 24:0), *cis*-monounsaturated fatty acids (MUFA) (16:1n-7, 16:1n-9, 17:1, 18:1n-5, 18:1n-7, 18:1n-9, 20:1, 22:1, 24:1), n-6 polyunsaturated fatty acids (PUFA) (18:2, 18:3, 20:2, 20:3, 20:4) and n-3 PUFA (18:3, 20:3, 20:5, 22:5, 22:6), long-chain n-6 PUFA (20:2, 20:3, 20:4), long-chain n-3 PUFA (20:3, 20:5, 22:5, 22:6), ruminant *trans* fatty acids (rTFA) (18:1n-7, CLA), and industrial *trans* fatty acids (iTFA) (16:1n-9, 18:1n-9, 18:2n-6, 18:3n-3).

### Statistical analysis

Cox proportional hazards regression using age as the underlying time metric with the subjects’ age at recruitment as the entry time and their age at cancer diagnosis (except for non-melanoma skin cancer), death, emigration or last complete follow-up, whichever occurred first, as the exit time was used to estimate the hazard ratio (HR) and 95% confidence interval (CI) for the association between dietary fatty acids and endometrial cancer risk. Intakes of fatty acids were log-transformed (in order to normalise the distribution) and divided into quintiles based on their distribution in all cohort women participants at baseline, setting women in the lowest category of fatty acids intake as the reference group. All models were stratified by the study center and age at enrolment (in one-year categories). The final multivariable model retained was adjusted for body mass index (BMI) (continuous), number of full term pregnancies (number of live born and/or still born children; 0, 1–2, 3–4; >4; missing), smoking status (never, former, current smokers), oral contraceptive or HRT use (never or ever), menopausal status at enrolment (premenopausal (women are considered premenopausal when they reported having had regular menses over the past 12 months or were younger than 46 years at recruitment); postmenopausal (women were considered postmenopausal when they reported not having had any menses over the past 12 months, reported having had a bilateral ovariectomy, or were older than 55 years); perimenopausal/unknown menopause (women were considered as perimenopausal when they were between age 46 and 55 years and had missing or incomplete questionnaire data), age at menarche (continuous) and total energy intake (continuous). Additional potential confounders (including history of breastfeeding (yes or no), physical activity (active or inactive), usual intake of alcohol (yes or no)) were not included in the final models as they did not alter the relative risk estimates by ˃10% (data not shown). In addition, mutual adjustment of fatty acids for each other did not modify the risk estimates (data not shown). Tests for trend were computed using the quintile specific median of each fatty acid. Stratified analysis by BMI (< 25 vs. ≥ 25 kg/m^2^), parity (nulliparous vs. parous), or menopausal status (pre vs. postmenopausal), and sensitivity analyses excluding the first 2 years of follow-up. All p for heterogeneity were > 0.05.

Due to the number of tests performed, q-values were calculated using the false discovery rate of the Benjamini-Hochberg procedure [22].

Additionally, associations between individual fatty acids intake (as continuous log-transformed variables) and endometrial cancer risk were investigated by their dietary sources grouping plant sources versus animal sources. The percentage of contribution to individual fatty acids intake was calculated for each food source based on the mean daily intake of dietary sources reported in the questionnaire.

All statistical analyses were carried out using STATA 14.0 (StataCorp, College Station, TX, USA). P-values below 0.05 were considered statistically significant.

## Results

Compared to the non-cases, endometrial cancer cases had higher BMI, were more likely to be nulliparous, post-menopausal, to have ever used HRT and to have a lower education status. They also used less oral contraceptives (Table 1).

**Table 1:** Characteristics of the study population

EPIC-wide study		
	Endometrial cancer cases	Non cases*
N = 299,318	n = 1,886	n = 297,432
Histological subtypes, number, (%**)		
Type I^§^	1690 (89.6)	-
Type II^§^	90 (4.7)	-
**Follow-up characteristics** Mean ± SD**		
Age at recruitment, years	54.9 ± 7.5	50.2 ± 9.9
Age at diagnosis, years	63.7 ± 8.1	-
Follow-up, years	8.8 ± 4.7	14.0 ± 3.8
Anthropometry Mean ± SD**		
Weight, kg	70.9 ± 13.9	65.3 ± 11.6
Height, cm	162.2 ± 6.6	162.3 ± 6.7
BMI, kg/m^2^	26.9 ± 5.3	24.8 ± 4.4
Obese (BMI ≥30 kg/m^2^), %**	24.2	12.1
**Reproductive and hormone factors**		
Number of full-term pregnancies#	1.9 ± 1.2	1.9 ± 1.2
Nulliparous, %	16.4	15.6
Ever use Oral contraceptives, %**		
Never	58.6	40.2
Ever	41.4	59.7
Ever use hormone replacement therapy##, %**		
Never	66.3	76.9
Ever	33.7	23.1
Ever breastfed#, %**		
No	28.7	27.9
Yes	71.3	72.1
Menopausal Status, %**		
Premenopausal	20.5	38.2
Post-menopausal	59.5	43.8
Perimenopausal	19.6	17.7
Age at menopause##	50.9 ± 4.1	49.3 ± 4.4
**Socio-economic status and lifestyle**		
Total energy intake, Kcal/day	1949.5 ± 539.8	1993.1 ± 546.1
Alcohol intake, %**		
None	18.5	16.4
< 5 g/day	32.7	31.9
5 to ≤ 14.9 g/day	28.2	30.1
15.0 to < 29.9 g/day	12.7	13.2
≥29.9 g/day	7.3	7.7
Education status, %		
None and primary school	33.8	27.8
Technical or professional and secondary school	44.1	45.0
Higher education	17.8	23.6
Physical activity status, %**		
Inactive	14.2	15.2
Moderately inactive	34.5	35.9
Moderately active	43.4	40.7
Active	7.9	8.2
Smoking status, %**		
Never	62.7	56.8
Former	21.7	22.9
Current **Other non-communicable diseases** number, (%**)	15.5	20.2
Diabetes	68 (4.1)	6149 (2.2)
Hyperlipidemia	205 (15.7)	30,481 (13.5)
Hypertension	452 (29.4)	48,832 (18.8)
Myocardial infarction	20 (1.2)	1754 (0.6)
Stroke	17 (1.1)	1783 (0.7)
**Dietary intake, (g/day)** Median (95%CI)**		
Dairy products	255.3 (245.7-265.2)	242.2 (241.4-242.9)
Fruits, nuts and seeds	199.6 (192.4-207.1)	190.6 (190.0-191.2)
Vegetables	168.7 (163.7-173.9)	178.1 (177.7-178.5)
Cereal and cereal products	173.1 (169.3-176.9)	182.5 (182.1-182.8)
Meat and meat products	71.3 (68.3–74.5)	61.3 (61.1–61.6)
Fish and shellfish	28.6 (27.2–29.9)	26.6 (26.5–26.7)
Egg and egg products	11.8 (11.3–12.4)	11.5 (16.7–18.3)
Fat	19.4 (18.8–20.1)	20.0 (19.9–20.1)
**Fatty acid intake** ^**###**^ **(g/day or mg/day)** Median (95%CI)**		
SFA (g/day)	23.7 (11.2–45.8)	24.9 (11.7–48.3)
*Cis* MUFA (g/day)	23.4 (11.3–45.2)	24.7 (12.1–48.5)
rTFA (mg/day)	22.9 (3.1-116.4)	27.9 (4.3-134.5)
iTFA (g/day)	1.2 (0.1–5.3)	1.2 (0.2–4.9)
*n*-6 PUFA (g/day)	11.0 (5.4–21.4)	11.4 (5.8–22.5)
n-6 linoleic acid (g/day)	10.9 (5.3–21.3)	11.38 (5.7–22.4)
n-6 γ-Linolenic acid (mg/day)	6.9 (1.9–22.5)	7.3 (1.8–24.3)
n-6 long-chain PUFA (mg/day)	23.7 (7.4–66.7)	24.2 (5.5–66.0)
n-3 PUFA (mg/day)	701.5 (254.0-1945.3)	667.2 (237.5-1913.2)
n-3 α-linolenic acid (mg/day)	379.4 (116.7-1263.6)	382.2 (117.5-1251.9)
n-3 long-chain PUFA (mg/day)	229.1 (28.8-1156.8)	198.1 (21.7-1051.9)

*Considered as non-cases at the most recent cancer endpoint and vital status update

**Continuous variables are presented as means and standard deviations (SD) or median (95%CI). Categorical variables are presented as percentages. Missing values were excluded from percentage calculations

^*§*^ *Type I included: adenocarcinoma (NOS), adenocarcinoma in adenomatous polyp, endometrioid adenocarcinoma (NOS), mucinous adenocarcinoma, mucin-producing adenocarcinoma, adenosquamous carcinoma or adenocarcinoma with squamous metaplasia. Type II included: squamous cell carcinoma (NOS), clear cell adenocarcinoma (NOS), mixed cell adenocarcinoma, serous cystadenocarcinoma (NOS) or papillary serous cystadenocarcinoma*

*#Among parous women*

*##Among postmenopausal women only*

*###Groupings of fatty acids are as described in the methods, assessment of dietary fatty acids intake*

**Fig. 1:**
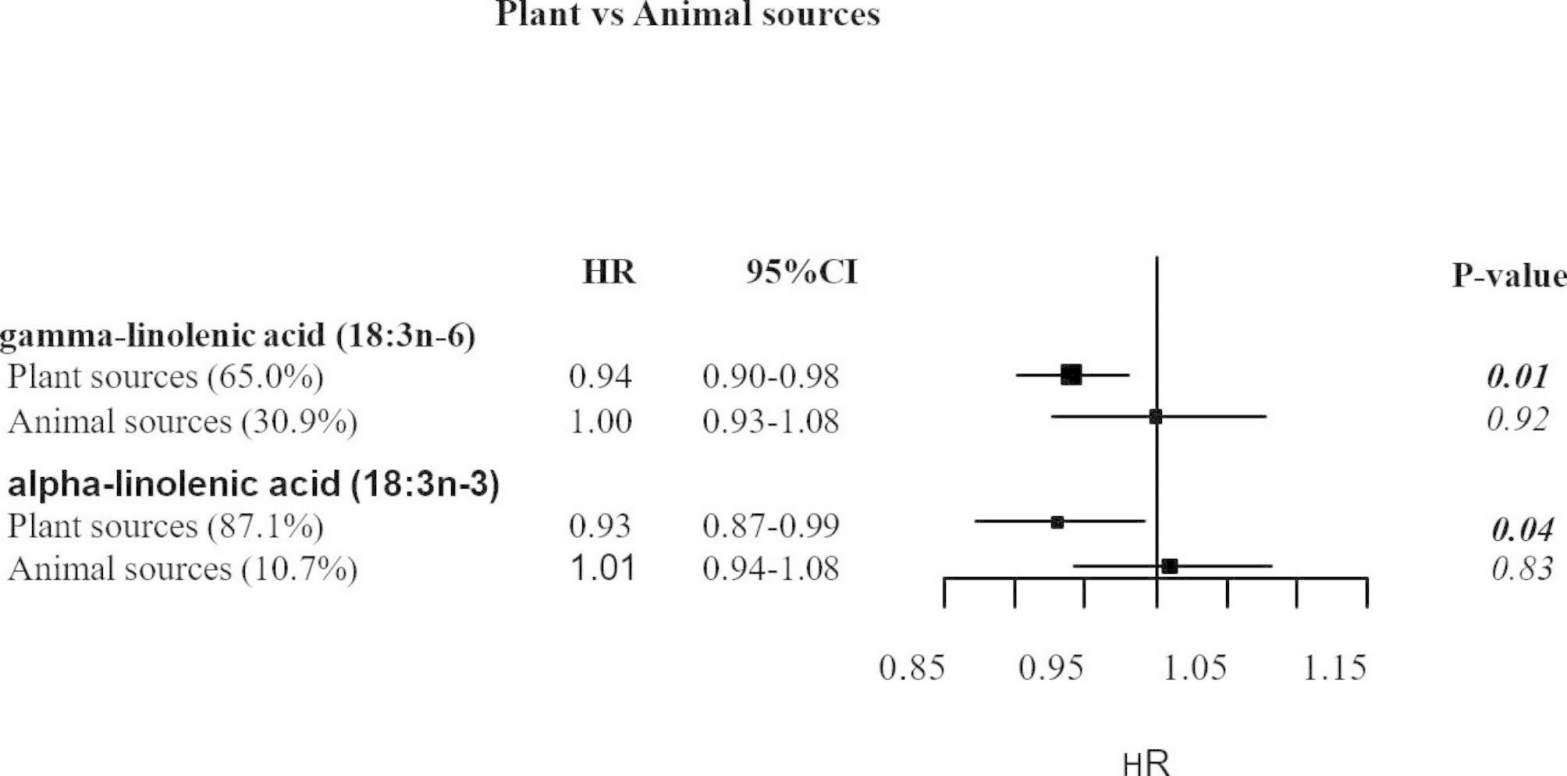
Associations between plant and animal sources of gamma- and alpha-linolenic acids with endometrial cancer risk *gamma-linolenic acid (18:3n-6)*: *The percentage of contribution next to the food sources was calculated for each food (sub-) group based on the mean daily intake reported in the dietary questionnaire. It represents the contribution of the corresponding source to the gamma-linolenic acid intake. Contribution of the plant sources (potatoes and other tubes (0.5%), vegetables (2%), fruit, nuts and seeds (0.2%), cereal and cereal products (14%), fat (19.7%), condiments and sauces (27.6%), soups and bouillons (0.3%) and miscellaneous (0.7%)) to γ-linolenic acid = 65.0% vs. animal sources (dairy products (6.6%), meat and meat products (13.7%), fish and shellfish (2.8%) and egg and egg products (7.8%)) = 30.9%* *alpha-linolenic acid (18:3n-3)*: *The percentage of contribution next to the food sources was calculated for each food (sub-) group based on the mean daily intake reported in the dietary questionnaire. It represents the contribution of the corresponding source to the alpha-linolenic acid intake. Contribution of the plant sources (potatoes and other tubes (0.3%), vegetables (0.3%),legumes (1.7%), cereal and cereal products (27.9%), fat (33.4%), sugar and confectionery (0.7%) non-alcoholic beverages (0.1%), condiments and sauces (22.4%) and soups and bouillons (0.3%)) to α-linolenic acid = 87.1% vs. animal sources (dairy products (3.8%), meat and meat products (3.5%), fish and shellfish (0.7%), egg and egg products (0.4%) and butter (2.3%)) = 10.7%* *HR = Hazard Ratio; CI = confidence interval. The multivariable model was adjusted for BMI (continuous), number of full-term pregnancies (number of live born and/or still born children; 0, 1–2, 3–4; >4; missing), smoking status (never, former, current), oral contraceptive or HRT use (never or ever), menopausal status at enrolment (premenopausal; postmenopausal; perimenopausal/unknown menopause), age at menarche (continuous) and total energy intake (continuous)*

Intake of n-6 γ-linolenic acid was inversely associated with endometrial cancer risk (HR comparing 5th with 1st quintile_Q5−Q1_=0.77, 95% CI = 0.64: 0.92, p_trend_=0.01, q-value = 0.15). This association was mainly driven by γ-linolenic acid derived from plant sources (contribution of vegetable sources intake to γ-linolenic = 65%, HR_per unit increment_=0.94, 95%CI= (0.90;0.98), p_trend_=0.01)) (Fig. 1**)**. An inverse association was also found between intake of n-3 α-linolenic acid from vegetable sources and endometrial cancer risk (contribution of vegetable sources intake to α-linolenic = 87.1%, HR_per unit increment_= 0.93, 95%CI = (0.87;0.99), p_trend_=0.04) (Fig. 1**)**.

No other statistically significant associations were identified between the other fatty acids (palmitic, stearic, oleic and linoleic acids) including the *trans* FA (iTFA or rTF) and endometrial cancer risk (Table 2).

**Table 2:** Association of estimated dietary intakes of fatty acids with endometrial cancer risk in the EPIC cohort

	Q1	Q2	Q3	Q4	Q5	p trend†	q trend§
	Reference						
**Total SFA** ^**a**^							
Mean Intake ± SD (g/d)	13.49 ± 2.79	19.88 ± 1.47	25.01 ± 1.53	31.12 ± 2.11	44.59 ± 9.16		
Cases/non-cases (n)	460/59,404	389/59,475	379/59,484	338/59,526	320/59,543		
h (95% CI) *	1.00	0.89 (0.77;1.03)	0.91 (0.77;1.06)	0.87 (0.73;1.05)	0.94 (0.75;1.18)	0.45	0.86
**Palmitic acid (16:0)**							
Mean Intake ± SD (g/d)	7.51 ± 1.45	10.77 ± 0.74	13.30 ± 0.75	16.25 ± 0.99	22.49 ± 4.15		
Cases/non-cases (n)	442/59,422	402/59,462	396/59,467	335/59,529	311/59,552		
h (95% CI)*	1.00	0.95 (0.82;1.10)	0.96 (0.81;1.12)	0.88 (0.73;1.06)	0.90 (0.71;1.14)	0.25	0.86
**Stearic acid (18:0)**							
Mean Intake ± SD (g/d)	3.10 ± 0.66	4.61 ± 0.34	5.79 ± 0.35	7.20 ± 0.48	10.24 ± 2.12		
Cases/non-cases (n)	420/59,444	395/59,469	388/59,475	365/59,499	318/59,594		
h (95% CI)*	1.00	0.96 (0.82;1.11)	0.91 (0.78; 1.11)	0.91 (0.76;1.09)	0.85 (0.68;1.07)	0.19	0.82
**Total** ***cis*** **-MUFA** ^**b**^							
Mean Intake ± SD (g/d)	13.79 ± 2.64	19.86 ± 1.40	24.77 ± 1.48	30.82 ± 2.13	44.61 ± 9.49		
Cases/non-cases (n)	437/59,427	404/59,460	403/59,460	359/59,505	283/59,580		
h (95% CI)*	1.00	1.02 (0.88;1.18)	1.09 (0.93;1.29)	1.06 (0.87;1.29)	0.99 (0.77;1.28)	0.72	0.96
**Oleic acid (18:1n-9)**							
Mean Intake ± SD (g/d)	12.72 ± 2.45	18.45 ± 1.33	23.14 ± 1.42	28.96 ± 2.04	42.32 ± 9.26		
Cases/non-cases (n)	448/59,417	402/59,461	391/59,472	365/59,499	280/59,583		
h (95% CI)*	1.00	0.99 (0.86;1.15)	1.04 (0.88;1.23)	1.06 (0.87;1.29)	0.97 (0.75;1.25)	0.74	0.96
**Total ruminant trans fatty acids** ^**d**^							
Mean Intake ± SD (mg/d)	6.00 ± 3.00	15.00 ± 3.00	29.00 ± 5.00	52.00 ± 8.00	120.00 ± 57.00		
Cases/non-cases (n)	493/59,372	362/59,502	386/59,476	330/59,534	315/59,548		
h (95% CI)*	1.00	0.95 (0.81;1.11)	1.10 (0.93;1.29)	1.02 (0.85;1.22)	1.13 (0.93;1.38)	0.22	0.86
**Total industrial trans fatty acids** ^**e**^							
Mean Intake ± SD (g/d)	0.30 ± 0.14	0.73 ± 0.12	1.20 ± 0.15	1.93 ± 0.28	4.19 ± 1.68		
Cases/non-cases (n)	358/59,506	356/59,508	369/59,494	391/59,473	412/59,451		
h (95% CI)*	1.00	1.12 (0.95;1.32)	1.08 (0.91;1.29)	1.08 (0.90;1.30)	1.05 (0.86;1.27)	0.92	0.98
**Elaidic acid (18:1n-9/12)**							
Mean Intake ± SD (g/d)	0.27 ± 0.13	0.69 ± 0.12	1.13 ± 0.15	1.85 ± 0.28	4.13 ± 1.68		
Cases/non cases (n)	357/59,507	353/59,511	364/59,499	398/59,466	414/59,449		
h (95% CI)*	1.00	1.12 (0.95;1.33)	1.09 (0.91;1.31)	1.11 (0.92;1.34)	1.06 (0.87; 1.30)	0.77	0.96
**Total** ***cis*** **n-6 PUFA** ^**f**^							
Mean Intake ± SD (g/d)	6.51 ± 1.17	9.21 ± 0.63	11.43 ± 0.67	14.20 ± 0.98	20.76 ± 4.83		
Cases/non-cases (n)	468/59,396	360/59,504	360/59,503	389/59,475	309/59,554		
h (95% CI)*	1.00	0.87 (0.75;1.01)	0.87 (0.74;1.02)	1.01 (0.85;1.20)	0.83 (0.67;1.01)	0.43	0.86
**Linoleic acid (18:2n-6)**							
Mean Intake ± SD (g/d)	6.48 ± 1.16	9.18 ± 0.63	11.40 ± 0.67	14.15 ± 0.97	20.70 ± 4.80		
Cases/non-cases (n)	468/59,396	358/59,506	364/59,499	386/59,478	310/59,553		
h (95% CI)*	1.00	0.86 (0.75;1.00)	0.88 (0.75;1.03)	1.00 (0.85;1.19)	0.83 (0.68;1.02)	0.46	0.86
**γ-linolenic acid (18:3n-6)**							
Mean Intake ± SD (mg/d)	2.48 ± 0.86	4.92 ± 0.66	7.38 ± 0.78	10.91 ± 0.38	21.79 ± 9.58		
Cases/non-cases (n)	388/59,495	393/59,454	414/59,461	378/59,474	313/59,548		
h (95% CI)*	1.00	0.92 (0.80;1.07)	0.97 (0.83;1.13)	0.91 (0.78;1.08)	0.77 (0.64;0.92)	**0.01**	0.15
**Total long-chain n-6 PUFA** ^**g**^							
Mean Intake ± SD (mg/d)	8.00 ± 3.00	17.00 ± 2.00	24.00 ± 2.00	34.00 ± 4.00	61.00 ± 24.00		
Cases/non-cases (n)	356/59,510	396/59,471	386/59,477	390/59,473	358/59,501		
h (95% CI)*	1.00	0.94 (0.81;1.10)	0.96 (0.81;1.12)	0.99 (0.84;1.17)	0.93 (0.77;1.11)	0.65	0.96
**Total** ***cis*** **n-3 PUFA** ^**h**^							
Mean Intake ± SD (g/d)	0.29 ± 0.08	0.49 ± 0.05	0.67 ± 0.06	0.93 ± 0.10	1.70 ± 0.61		
Cases/non-cases (n)	350/59,514	372/59,492	341/59,522	395/59,469	428/59,435		
h (95% CI)*	1.00	1.00 (0.85;1.17)	0.91 (0.77;1.08)	0.97 (0.81;1.15)	0.91 (0.75;1.10)	0.33	0.86
**α-linolenic acid (18:3n-3)**							
Mean Intake ± SD (g/d)	0.15 ± 0.05	0.27 ± 0.03	0.38 ± 0.04	0.56 ± 0.07	1.10 ± 0.44		
Cases/non-cases (n)	367/59,497	405/59,461	379/59,482	350/59,515	385/59,477		
h (95% CI)*	1.00	1.08 (0.93;1.26)	1.05 (0.89;1.23)	0.96 (0.81;1.14)	0.94 (0.78;1.14)	0.27	0.86
**Total long-chain n-3 PUFA** ^**i**^							
Mean Intake (mg/d)	40.00 ± 21.00	115.00 ± 21.00	198.00 ± 27.00	338.00 ± 61.00	933.00 ± 609.00		
Cases/non-cases (n)	333/59,531	320/59,545	363/59,499	401/59,463	469/59,394		
h (95% CI)*	1.00	0.91 (0.77;1.07)	0.95 (0.80;1.12)	0.95 (0.80;1.13)	0.95 (0.79;1.15)	0.84	0.96
**Ratio n-6/n-3 PUFA**							
Mean Intake ± SD	7.76 ± 2.29	13.06 ± 1.24	17.47 ± 1.34	23.16 ± 2.07	39.06 ± 28.95		
Cases/non-cases (n)	491/59,373	419/59,445	334/59,529	334/59,530	308/59,555		
h (95% CI)*	1.00	1.11 (0.96;1.28)	0.96 (0.81;1.13)	1.03 (0.86;1.22)	1.04 (0.86;1.24)	0.98	0.98

*HR = hazard ratio; CI = confidence interval*

† *P or q values < 0.05 are shown in boldface type*

§ *Value for FDR (False Discovery Rate) correction*

* Stratified by study center and age (in one-year categories), andadjusted for BMI (continuous), number of full-term pregnancies (number of live born and/or still born children; 0, 1–2, 3–4; >4; missing), smoking status (never, former, current), oral contraceptive or HRT use (never or ever), menopausal status at enrolment (premenopausal; postmenopausal; perimenopausal/unknown menopause), age at menarche (continuous) and total energy intake (continuous)

^*a*^ *Total SFA included 4:0, 6:0, 8:0, 10:0, 12:0, 14:0, 15:0, 16:0, 17:0, 18:0, 20:0, 22:0, 24:0;* ^*b*^ *Odd chain fatty acids included 15:0, 17:0;* ^*c*^ *Total cis MUFA included 16:1n-7, 16:1n-9, 17:1, 18:1n-5, 18:1n-7, 18:1n-9, 20:1, 22:1, 24:1;* ^*d*^ *Total trans ruminant fatty acids included 18:1n-7t, CLA;* ^*e*^ *Total trans industrial fatty acids included 16:1n-9t, 18:1n-9t, 18:2n-6tt, 18:3n-3ttt;* ^*f*^ *Total n-6 PUFA included 18:2, 18:3, 20:2, 20:3, 20:4;* ^*g*^ *Total long-chain n-6 PUFA included 20:2, 20:3, 20:4;* ^*h*^ *Total n-3 PUFA included 18:3, 20:3, 20:5, 22:5, 22:6;* ^*i*^ *Total long-chain n-3 PUFA included 20:3, 20:5, 22:5, 22:6;* ^*j*^ *Total cis-PUFA included total n-6 PUFA and total n-3 PUFA*

Finally, the association between fatty acids and endometrial cancer did not vary according to histological subtypes of endometrial cancer (type I vs. type II). No substantial difference in the risk estimate was shown in the stratified analysis by BMI (< 25 vs. ≥ 25 kg/m^2^), parity (nulliparous vs. parous), or menopausal status (pre vs. postmenopausal), and in the sensitivity analyses excluding the first 2 years of follow-up. In all stratified analyses, no significant association was reported between fatty acids (grouped or individual) and endometrial cancer risk (data not shown). All p for heterogeneity were > 0.05.

## Discussion

In this large-scale prospective analysis, an inverse association between the consumption of n-6 γ-linolenic acid and n-3 α-linolenic acid and endometrial cancer risk was found and this association was mainly driven by the vegetable sources of these two fatty acids. These associations did not vary according to histological subtypes of endometrial cancer.

Besides γ-linolenic acid and α-linolenic acid from vegetable sources, no significant association between any other dietary fatty acids and endometrial cancer was reported in this study. Our results align with results from the NHS and NHSII studies [12] but not with findings from a previous analysis within the EPIC study, which reported an inverse association between total fat intake, total MUFA and endometrial cancer [12]. This is probably due to the fact that in the current EPIC analysis, in addition to having a longer follow-up and more endometrial cancer cases, a better separation between *cis* and *trans* MUFA isomers was available after the USDA matching (the mean of total MUFA intake in the previous EPIC analysis was 29 g/day vs. 24 g/day of *cis*-MUFA in this current analysis) [18].

The overall inverse association between γ-linolenic acid and endometrial cancer risk was mainly driven by its vegetable sources, mainly from cereal and cereal products (14%), fat (19.7%) and condiments and sauces (27.6%). In addition, the inverse association between endometrial cancer and n-3 α-linolenic acid from vegetable sources (mainly cereal and cereal products (27.9%), fat (33.4%) and condiments and sauces (22.4%)) was not observed for n-3 α-linolenic acid from animal sources.

Our study is the first to investigate the associations between animal and plant sources of fatty acids and endometrial cancer risk. MUFA from plant sources also have an aded value in decreasing overall mortality including cardiovascular and cancer mortality, as recently reported in the NHS and Health Professionals Follow-Up Study (HPFS) [23]. Our data suggest that vegetable sources of γ-linolenic acid and α-linolenic acid may exert a protective effect on endometrial cancer risk. The mechanisms underlying these inverse associations might be explained by the fact that γ-linolenic acid and α-linolenic were both reported to induce apoptosis in an experimental study on cancer cell lines. However, this in vitro analysis has also reported additional differential antitumor effects of γ-linolenic acid and α-linolenic acid. α-linolenic was reported to affect some cellular pathways, particularly the mitochondrial protein import pathway and the cycle of citric acid whereas γ-linolenic acid has no specific actions on these pathways [24]. Besides their direct effects on cancer development, anti-carcinogenic components of the vegetable sources of these fatty acids (mainly nuts and seeds) which are rich in vitamins, minerals and a range of active metabolites such as phenolic acids, phytosterols, carotenoids, and polyphenolic compounds, might also contribute to this inverse association [25]. Moreover, the mixture of all these components or the called “matrix effect” might explain this association and not necessarily each component by itself [26], the modulation of steroid hormone concentrations and metabolism, the activation of antioxidant mechanisms, the regulation of detoxification enzymes, and/or the stimulation of the immune system [27].

In this study, no significant association was found between n-6 and n-3 PUFA overall, or long-chain PUFA, and endometrial cancer risk. However, these two families are known to playing a significant role in cancer by generating modulatory molecules for inflammatory responses, including eicosanoids (prostaglandins and leukotrienes), and cytokines (interleukins) and by affecting the gene expression of several bioactive molecules. Linoleic acid is an essential FA, derived only from diet and mainly from seeds, nuts vegetable oils (safflower oil, maize oil, sunflower oil and soybean oil), meat and eggs. γ-linolenic acid is derived from linoleic acid, (by Δ6-desaturase) and can be prolongated by the enzyme elongase 5 to dihomo-γ-linolenic acid (20:3n-6; DGLA) [28]. After this, DGLA go through oxidative metabolism by cyclooxygenases and lipoxygenases to generate anti-inflammatory eicosanoids (prostaglandins of series 1 and leukotrienes of series 3) [29, 30]. With these same series of enzymes, n-3 α-linolenic acid (the essential n-3 PUFA derived mainly from seeds (flaxseeds and flaxseed oils) and nuts), competing with linoleic and γ-linolenic acid, is converted into long-chain fatty acids (LC-PUFA): eicosapentaenoic acid (20:5n-3; EPA) and docosahexaenoic acid (22:6n-3;DHA). Found in oily fish and fish supplements, these fatty acids can increase anti-inflammatory and inflammation resolving mediators called resolvins, protectins and maresins. They can also inhibit many inflammation facets including leucocyte chemotaxis, adhesion molecule expression and leucocyte–endothelial adhesive interactions, production of eicosanoids like prostaglandins and leukotrienes from the n-6 arachidonic acid, and production of pro-inflammatory cytokines [28].

Inflammation has been linked to endometrial cancer in several cohort and case-control studies [31–33]. LC-PUFA (EPA and DHA), which are suggested to be anti-inflammatory (as described above), could potentially reduce endometrial cancer risk [34]. However, epidemiological results in this field are inconclusive. One Japanese case-control study reported a lower risk of endometrial cancer in association with higher fish consumption (significant inverse association with 16.02 g/1000Kcal of the mean intake of fish in the Japanese study vs. 13.80 g/1000Kcal of fish intake in this current study) [35], whereas several other case-control and cohort studies reported no statistically significant associations [15, 36–38]. Similarly, our data showed no significant association between n-3 LC-PUFA and endometrial cancer risk. This is probably due to the fact that the mean intake of EPA (57 mg/day) and DHA (97 mg/day) in our study was lower than the one recommended in the US (> 500 mg/day EPA + DHA) and in Europe (250 mg/day EPA + DHA) [39]. In addition, we didn’t have data regarding PUFA supplementation to consider. Further studies are needed to clarify the potential association between n-3 LC-PUFA, fish intake and endometrial cancer risk.

iTFA consumption is associated with an increased risk of all-cause mortality [40], and the WHO supports actions to eliminate these fatty acids from the diet [41]. Epidemiological data on the association between iTFA and cancer risk are few [42]. However, in agreement with other studies [14, 15], we did not find any significant association between iTFA and endometrial cancer risk. Contrary to the positive association that we reported with breast and ovarian cancer development in the EPIC cohort [43, 44], this present study suggests that iTFA from industrial processes are not associated with endometrial cancer development.

The strengths of our study are several including its prospective design, the very large number of incident endometrial cancer cases and our ability to separate *n*-6 and *n*-3 *cis* PUFA isomers. The main limitation of this study is the single collection at baseline of questionnaire dietary data potentially causing random measurement errors and failing to reflect long-term habits. These biases may underestimate true associations. In addition, no information was provided regarding PUFA supplementation, so our analyses were limited to dietary intake only. Another limitation is that the biomarkers of fatty acids were not available in this study; their availability would have allowed a complementary assessment for the associations between fatty acids and endometrial cancer risk. In addition, results regarding analysis by histological subtypes were not conclusive and probably underpowered due to the small sample size of type II tumors.

## Conclusion

To our knowledge, this is the first large-scale study to scrutinize the effects of dietary sources of fatty acids (animal or plant)- on endometrial cancer risk. Our findings in EPIC showed that plant sources of the essential n-6 and n-3 PUFA were inversely associated with endometrial cancer development, suggesting that the dietary source of fatty acids (animal versus plant) may be important when investigating the association between fatty acids and cancer risk.

## Data Availability

For information on how to submit an application for gaining access to EPIC data and/or biospecimens, please follow the instructions at http://epic.iarc.fr/access/index.php.

## Abbreviations

CI: Confidence Interval
DGLA: Dihomo-γ-Linolenic Acid
DHA: Docosahexaenoic acid
ENDB: EPIC Nutrient Database
EPA: Eicosapentaenoic Acid
EPIC: European Prospective Investigation into Cancer and Nutrition
HPFS: Health Professionals Follow-Up Study
HR: Hazard Ratio
HRT: Hormonal Replacement Therapy
IARC: International Agency for Cancer Reasearch
iTFA: industrial *Trans* fatty acids
LCPUFA: Long Chain Polyunsaturated Fatty Acids
MUFA: Monosaturated Fatty acids
NHS: Nurses’ Health Study
NNDSR: National Nutrient Database for Standard Reference of the United States
PUFA: Polyunsaturated Fatty Acids
rTFA: ruminant *Trans* Fatty Acids
SFA: Saturated Fatty acids
USDA: United States Department of Agriculture
WCRF: World Cancer Research Fund
WHO: World Health Organization
